# Gut microbiota of old mice worsens neurological outcome after brain ischemia via increased valeric acid and IL-17 in the blood

**DOI:** 10.1186/s40168-023-01648-1

**Published:** 2023-09-12

**Authors:** Xianzhang Zeng, Jun Li, Weiran Shan, Zhongmeng Lai, Zhiyi Zuo

**Affiliations:** 1 Department of Anesthesiology, University of Virginia, 1 Hospital Drive, PO Box 800710, Charlottesville, VA 22901 USA; 2 Department of Anesthesiology, Second Affiliated Hospital, Harbin Medical University, Harbin, 150001 Heilongjiang People’s Republic of China; 3 Department of Anesthesiology, Fujian Medical University Union Hospital, 29 Xin-Quan Road, Fuzhou, 350001 People’s Republic of China; 4 Departments of Neuroscience and Neurosurgery, University of Virginia, Charlottesville, VA 22901 USA

**Keywords:** Age, Focal brain ischemia, Gut microbiota, Interleukin-17, Mice, Valeric acid

## Abstract

**Background:**

Aging is a significant risk factor for ischemic stroke and worsens its outcome. However, the mechanisms for this worsened neurological outcome with aging are not clearly defined.

**Results:**

Old C57BL/6J male mice (18 to 20 months old) had a poorer neurological outcome and more severe inflammation after transient focal brain ischemia than 8-week-old C57BL/6J male mice (young mice). Young mice with transplantation of old mouse gut microbiota had a worse neurological outcome, poorer survival curve, and more severe inflammation than young mice receiving young mouse gut microbiota transplantation. Old mice and young mice transplanted with old mouse gut microbiota had an increased level of blood valeric acid. Valeric acid worsened neurological outcome and heightened inflammatory response including blood interleukin-17 levels after brain ischemia. The increase of interleukin-17 caused by valeric acid was inhibited by a free fatty acid receptor 2 antagonist. Neutralizing interleukin-17 in the blood by its antibody improved neurological outcome and attenuated inflammatory response in mice with brain ischemia and receiving valeric acid. Old mice transplanted with young mouse feces had less body weight loss and better survival curve after brain ischemia than old mice transplanted with old mouse feces or old mice without fecal transplantation.

**Conclusions:**

These results suggest that the gut microbiota-valeric acid-interleukin-17 pathway contributes to the aging-related changes in the outcome after focal brain ischemia and response to stimulus. Valeric acid may activate free fatty acid receptor 2 to increase interleukin-17.

**Supplementary Information:**

The online version contains supplementary material available at 10.1186/s40168-023-01648-1.

## Highlights

• Gut microbiota of old mice worsens ischemic brain injury and inflammation

• Valeric acid contributes to old mouse gut microbiota effects on the outcome of stroke

• Interleukin-17 contributes to old mouse gut microbiota effects on stroke outcome

• Gut microbiota-valeric acid-free fatty acid receptor 2-interleukin-17 pathway modulates aging-related response to stimulus

Video Abstract

**Supplementary Information:**

The online version contains supplementary material available at 10.1186/s40168-023-01648-1.

## Introduction

Stroke is a leading cause of death and morbidity in the world and often occurs in elderly patients [1, 2]. The outcome of ischemic stroke in elderly patients is worse than that in young patients [3, 4]. A similar situation has been shown in animal studies [5, 6]. Multiple factors may contribute to this phenomenon. For example, brain immune cells in old rodents may be in primed status and can have an exaggerated response to stimulation to produce proinflammatory cytokines that are known to worsen neurological outcome after brain ischemia [7, 8].

Studies have shown that gut microbiota can modulate inflammatory responses in the brain after brain ischemia [9, 10]. Antibiotics-induced changes in the gut flora provide neuroprotection against brain ischemia in mice [9]. A recent study has shown that aging-related changes in gut microbiota may influence the outcome of experimental stroke in mice [11]. However, the mechanisms for this effect are not defined. Consistent with these experimental stroke findings, stroke patients with significant gut dysbiosis may have a worsened neurological outcome [12], suggesting a potential role of gut microbiota in determining stroke outcome in humans.

Gut microbiota can produce multiple metabolites. Among them, short-chain fatty acids (SCFA) are one of the major types of metabolites and can regulate inflammatory responses [13, 14], a process that affects neurological outcome after brain ischemia [7, 8]. Previous studies have shown that SCFAs are decreased with aging in the feces [11, 15]. A recent study has shown that stroke patients have changes in SCFA concentrations in their feces [16]. However, whether SCFAs are involved in aging-related changes in brain ischemic tolerance and how SCFAs affect stroke outcome is not known.

Interleukin (IL)-17 is a proinflammatory cytokine. It is produced from a group of T helper cells and can induce the production of chemokines that recruit immune cells to the site of inflammation and facilitate the production of other proinflammatory cytokines, such as IL-6 and IL-1β [17, 18]. A previous study has shown that the decrease of IL-17-positive T helper cells may contribute to the neuroprotection induced by antibiotics-caused gut floral changes [9].

Our study was designed to determine whether aging-related changes in gut microbiota contribute to the worsened neurological outcome after brain ischemia and whether SCFAs and IL-17 mediate this gut microbiota effect. Our results suggest that a novel pathway, gut microbiota-valeric acid-free fatty acid receptor (FFAR) 2-IL-17, mediates increased inflammatory response to brain ischemia and worsened neurological outcome after ischemic stroke in old mice.

## Methods and materials

### Animals and experimental design

Eight-week-old C57BL/6J male mice (328 mice in total) were purchased from Charles River (Wilmington, MA, USA) and 18- to 21-month-old male C57BL/6J mice (66 mice in total) were provided by the National Institutes of Health (Bethesda, MD). They were maintained in the vivarium under pathogen-free conditions (23 ± 2°C; 12-h light/dark cycle) with free access to food and water and housed 3 to 5 mice per cage before and during the experiments. All experimental procedures were approved by the Institutional Animal Care and Use Committee of the University of Virginia (Charlottesville, VA, USA). All animal experiments were performed in accordance with the National Institutes of Health Guide for the Care and Use of Laboratory Animals (NIH publications number 80–23) revised in 2011.

**Fig. 1:**
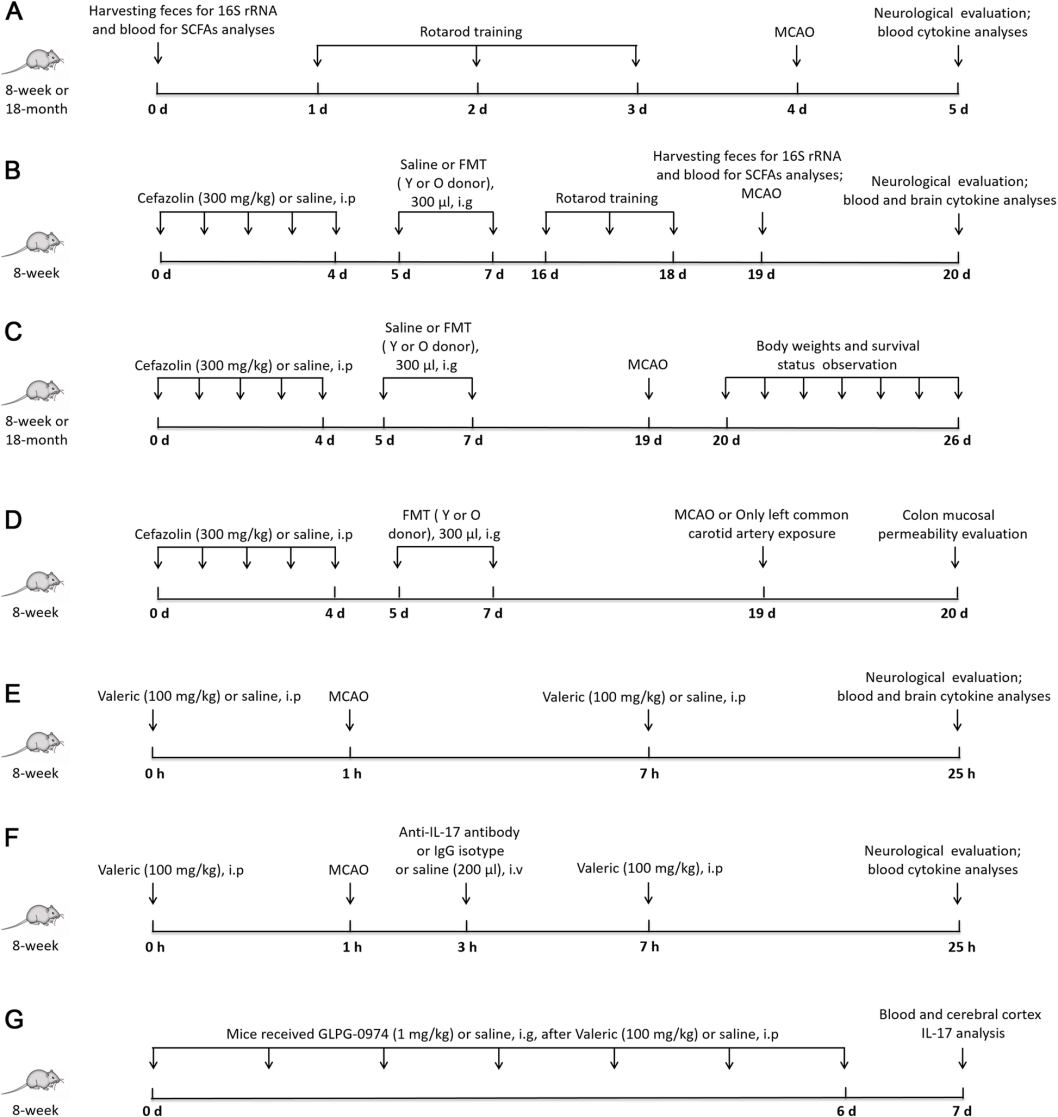
Diagrammatic presentation of experiments. O old, Y young, MCAO middle cerebral artery occlusion, FMT fecal microbiota transplant

In the first experiment (Fig. 1A), 8-week and 18-month-old C57BL/6J male mice were subjected to the left middle cerebral artery occlusion (MCAO). Blood and feces from mice of these two ages without MCAO were harvested for analyses of SCFAs in the blood and 16S rRNA in the feces. Neurological outcome and cytokines in the blood were evaluated 24 h after MCAO.

To explore the effects of gut microbiota of young (8 weeks) or old mice (18 ~ 20 months) on stroke, 8-week-old C57BL/6J male mice were randomly divided into four groups (Fig. 1B): control group that was treated with normal saline by intraperitoneal injection daily for 5 days and then normal saline by gastric gavage once a day for 2 days, antibiotic group that was treated with cefazolin 300 mg/kg daily by intraperitoneal injection for 5 consecutive days and then normal saline by gastric gavage once a day for 2 days, old mouse fecal transplantation (Young-oFMT) group that was treated with cefazolin 300 mg/kg daily by intraperitoneal injection for 5 consecutive days and then 300 μl fecal solution from old mice by gastric gavage once a day for 2 days, and young mouse fecal transplantation (Young-yFMT) group that was treated with cefazolin 300 mg/kg daily by intraperitoneal injection for 5 consecutive days and then 300 μl fecal solution from young mice by gastric gavage once a day for 2 days. Gastric gavage occurred on day 1 and day 3 after the last dosage of cefazolin. Feces and blood were harvested 14 days after the first gastric gavage for 16S rRNA and SCFA analyses. Mice were subjected to MCAO 2 weeks after the first gastric gavage. The neurological outcome was evaluated 24 h after MCAO. Blood and brain were harvested for cytokine analyses 24 h after MCAO in another cohort of mice. The third cohort of the same 4 groups of 8-week-old C57BL/6J male mice (control group, antibiotic group, Young-oFMT, and Young-yFMT) were used to determine the body weights and survival status after MCAO (Fig. 1C). Similarly, 18-month-old C57BL/6J male mice were randomly divided into four groups: control group, antibiotic group, old mouse fecal transplantation group (Old-oFMT), and young mouse fecal transplantation group (Old-yFMT). The treatments of these four groups were the same as described above for 8-week-old C57BL/6J mice. Body weights and survival status were observed after MCAO (Fig. 1C).

To determine the effects of gut microbiota of young or old mice on the colon mucosal permeability after stroke, 8-week-old C57BL/6J male mice were randomly divided into four groups (Fig. 1D): old mouse fecal transplantation-sham group that received old mouse fecal transplantation and left common carotid artery exposure but without the induction of MCAO, young mouse fecal transplantation-sham group that received young mouse fecal transplantation and left common carotid artery exposure but without the induction of MCAO, and old mouse fecal transplantation-MCAO group and young mouse fecal transplantation-MCAO group. Colon mucosal permeability was tested 24 h after ischemic reperfusion.

To investigate the effects of valeric acid on stroke, 8-week-old C57BL/6J male mice were randomly divided into two groups (Fig. 1E): saline-stroke group that received intraperitoneal 100 μl normal saline 1 h before MCAO and 6 h after MCAO and valeric-stroke group that received intraperitoneal 100 mg/kg in 100 μl valeric sodium (catalog number: V091420, LOT number: 1-SHE-30–1, Toronto Research Chemicals, Inc., Toronto, ON, Canada) 1 h before MCAO and 6 h after MCAO. Neurological outcome was evaluated 24 h after MCAO. Blood and brain were harvested for cytokine analyses. A pilot experiment was performed to determine the dosage of valeric acid used in this study. Eight-week-old mice were divided into two groups: the Y-saline group that received two intraperitoneal 100 μl normal saline injections at an interval of 7 h and the Y-Valeric group that received two intraperitoneal 100 mg/kg in 100-μl valeric sodium injections at an interval of 7 h. One group of 18-month-old mice that was labeled O-saline received two intraperitoneal 100-μl normal saline injections at an interval of 7 h. The blood was harvested 3 h after the second injection of valeric sodium or normal saline to measure valeric acid.

To test the role of IL-17 in valeric acid-worsened neurological outcome after MCAO, 8-week-old C57BL/6J male mice that received 2 doses of valeric acid as described above were randomly divided into three groups (Fig. 1F): the saline control group that received intravenous 200-μl normal saline 2 h after the initiation of MCAO, the IgG isotype control group that received intravenous 100-μg mouse IgG1 kappa isotype control (catalog number:14–4714-85, LOT number: 2087674, Thermo Scientific, Rockford, IL, USA) in 200 μl 2 h after the initiation of MCAO, and the anti-IL-17 antibody group that received intravenous 100-μg mouse monoclonal anti-IL-17 antibody (catalog number:16–7173-85, LOT number: E06909, Thermo Scientific, Rockford, IL, USA) in 200 μl 2 h after the initiation of MCAO. Neurological outcome was evaluated 24 h after MCAO. The blood was harvested for cytokine analyses.

To determine whether valeric acid increased IL-17 via SCFA receptors, 8-week-old male mice were randomly divided into four groups (Fig. 1G): the saline control group that received intraperitoneal 100-μl normal saline for 7 days; the valeric acid group that received intraperitoneal 100 mg/kg in 100-μl valeric sodium for 7 days; the valeric acid plus antagonist group that received 1 mg/kg GLPG-0974 (catalog number: SML2443, Sigma-Aldrich, Inc. St. Louis, MO, USA), a free fatty acid receptor 2 (FFAR2) antagonist [19], by gastric gavage immediately after each of the valeric acid intraperitoneal injections for 7 days; and the antagonist control group that received 1 mg/kg GLPG-0974 by gastric gavage, once a day, for 7 days. The blood and cerebral cortex were collected for IL-17 analysis.

### Fecal transplantation

Fresh fecal pellets collected within 30 min after bowel movement from old (18 to 21 months old) or young (8 weeks old) mice were diluted with 100 mg/ml sterile saline. All fecal pellets of old mice were from the same eleven old mice and re-suspended together in saline. These donors also provided feces individually for gut microbiome profiling as the old mouse group. However, the feces of different sets of young mice (10 mice for each set) were harvested for transplantation to keep the donor age at 8 to 10 weeks. These donors (old mice or young mice) were housed together at 3 to 5 mice of similar ages per cage. Briefly, the fecal matter was vortex-mixed for 5 min and then passed through 70-μm nylon cell strainer (catalog number: 08–771-2, Fisher Scientific, San Diego, CA, USA) to remove undigested food and smaller particulate materials. To facilitate the colonization of the transplanted microbiota, recipient mice received an intraperitoneal injection of 300 mg/kg cefazolin once daily for 5 consecutive days to eliminate the original gut microbiota in the recipients. This regimen reduced the gut microbiota in the colon to an almost undetectable level by 16S rRNA analyses in our previous study [20]. The recipient mice received 300-μl corresponding fecal solution by gastric gavage at 24 h and 72 h after the last dose of cefazolin. A volume at 10 ml/kg and 20 ml/kg as an ideal and maximal volume, respectively, for gastric gavage has been recommended for mice [21] and a volume at 500 μl was used in our previous study [22]. An interval of 24 h between the last dose of cefazolin and receiving fecal solution was used because an interval of 1 to 2 days is commonly used in gut microbiota transplant studies [22–24]. A very small amount of cefazolin may remain in the mice 24 h later because its plasma half-life in rodents is 0.5 h [24]. Transplanted mice were maintained for 2 weeks after fecal transplantation before they were subjected to MCAO.

### Middle cerebral artery occlusion

As we described previously [25, 26], left MCAO with intravascular suture technique was performed in mice. Briefly, mice were anesthetized with isoflurane and the rectal temperature was maintained at 37 ± 0.5 °C by the feedback-controlled heating system. A monofilament nylon suture (catalog number: 1622-A1, Beijing CiNontech Co. Ltd., Beijing, China) was used to induce the MCAO via the external carotid artery to the left internal carotid artery until slight resistance was felt. Mice were re-anesthetized by isoflurane and the sutures were removed at 60 min (only for the set of experiments of old mice with fecal transplantation; this selection was because of a high mortality rate after a longer MCAO) or 120 min (for all other sets of experiments) after the onset of MCAO.

### Evaluation of motor co-ordination, neurological deficit scores, and infarct volumes

The infarct volumes were assessed 24 h after MCAO by a blinded investigator after 2,3,5-triphenyltetrazolium chloride staining [25, 26]. Briefly, mice were anaesthetized deeply with 5% isoflurane and transcardiacally perfused by saline. Brains were cut into 6 2-mm-thick coronal slices to evaluate the infarct volume. The average infarct area of the rostral and caudal sides of each slice was calculated. The infarct volume of the brain slice was calculated by multiplying the average infarct area of the slice by the thickness of the slice. The sum of infarct volumes of all brain slices from the mouse was considered as the total infarct volume of the brain. To correct the cerebral edema and differential shrinkage resulting from brain ischemia and tissue processing, the corrected brain infarct volume was calculated: corrected brain infarct volume in percentage = [right hemisphere volume—(left hemisphere volume—left infarction volume)] × 100/right hemisphere volume.

Neurological deficit scores were tested 24 h after the stroke by a blinded investigator [25, 26]. Briefly, mice were scored as follows: zero, no apparent deficits; one, failure to extend right forepaw fully; two, decreased grip of the right forelimb; three, spontaneous movement in all directions, contralateral circling only if pulled by the tail; four, circling or walking to the right; five, walking only if stimulated; six, unresponsiveness to stimulation and with depressed level of consciousness; and seven, dead.

Rotarod test was performed to evaluate the motor coordination as we described before [25, 26]. All mice received the training for three continuous days before the induction of brain ischemia. The formal test was performed 24 h before and 24 h after the MCAO. Mice were placed on a rotarod with the speed accelerated from 4 to 40 rpm within 5 min. The latency and the speed were recorded when the tested mice fell off the rod. Each mouse was tested five times. The speed-latency index, which is latency (s) × speed (rpm), was calculated. The ratio of mean index of the five trials obtained before MCAO and after MCAO was calculated to reflect the change in coordinate function of each mouse.

### In vivo colon mucosal permeability assay

Colon mucosal permeability was measured under the in vivo condition using Evans blue as described before [27]. Mice were placed in a supine position on a heating pad and a laparotomy was performed under isoflurane anesthesia with spontaneous breathing. The cecum was dissociated and placed outside the abdominal cavity. A small hole was cut in the cecum. A small polyethylene tube (G18) was inserted into the rectum through the anus and secured by a ligature. The colon was gently flushed with phosphate buffered saline (PBS) via this tube until all feces were rinsed out. The proximal and distal colon were ligated and 1 ml of 1.5% Evans blue (catalog number: E2129-10G, Sigma-Aldrich, St. Louis, MO, USA) in PBS was instilled into the colon. The colon was rinsed 30 min later with PBS until the perianal washout was clear. Then, the mice were sacrificed under deep anesthesia and the colon was removed rapidly. The dissected colon was opened and rinsed again with 3 ml PBS, followed by 5 ml 6 mM N-acetylcysteine (catalog number: PHR1098-1G, Sigma-Aldrich, St. Louis, MO, USA) to eliminate any unabsorbed Evans blue in the colonic mucus. The colon was weighed and then placed in 1 ml formamide (catalog number: F9037-100ML, Sigma-Aldrich, St. Louis, MO, USA) at 50 °C for 24 h to extract the Evans blue dye. The dye concentration in the supernatant was measured spectrophotometrically at 655 nm and given as microgram per gram colonic tissue.

### Gut microbiota profiling

Fresh feces from mice were collected for gut microbiota profiling. Mice were placed individually in an autoclaved cage for collecting feces and allowed to defecate freely. Feces were collected immediately in the sterile eppendorf tubes on ice and then stored at − 80°C until further processing. Bacteria DNA was extracted with Power Lyzer Power soil DNA isolation kit (catalog number: 12855–100, QIAGEN, Germantown, MD, USA) according to our previous protocols [20]. Library preparation and sequencing were performed by LC Sciences (Houston, TX). Primers used were as follows: 341F: 5'-CCTACGGGNGGCWGCAG-3' and 805R: 5'-GACTACHVGGGTATCTAATCC-3', which were designed to target the V3 and V4 regions of 16S rDNA to generate an amplicon of about 465 bp in size. The amplified library is sequenced on a NovaSeq 6000, 2 × 250 bp (NovaSeq 6000 SP Reagent Kit, 500 cycles). Paired-end reads was assigned to samples based on their unique barcode and truncated by cutting off the barcode and primer sequence. Paired-end reads were merged using FLASH. Quality filtering on the raw reads was performed under specific filtering conditions to obtain the high-quality clean tags according to the fqtrim (v0.94). Chimeric sequences were filtered using Vsearch software (v2.3.4). After dereplication using DADA2, feature table and sequence were obtained. Alpha diversity and beta diversity were calculated by being normalized to the same sequences randomly. Feature abundance was normalized using relative abundance of each sample according to SILVA (release 132) classifier. Alpha diversity including Chao1 index, observed_otus index, and Shannon index was used for the analysis of species diversity in a single sample. Principal coordinate analysis (PCoA) and analysis of similarity (ANOSIM) using Jaccard distance metrics were used for Beta diversity analysis to compare microbial compositions among samples. The relative abundance of taxa within gut microbiota at family level was compared among groups. The gut microbiota data analysis was performed by LC Sciences.

### 16S rRNA quantification

Fecal bacterial DNA (25 ng) was used for 16S rRNA PCR to amplify the V3 and V4 region by using the following primers: F: 5’-TCGTCGGCAGCGTCAGATGTGTATAAGAGACAGCCTACGGGNGGCWGCAG-3’ and R: 5’-GTCTCGTGGGCTCGGAGATGTGTATAAGAGACAGGACTACHVGGGTATCTAATCC-3’. The reaction settings were 95°C for 3 min, 25 cycles of 95°C for 30 s, 55°C for 30 s, 72°C for 30 s, and 72°C for 5 min and then stored at 4°C [20]. The amplicons were separated in 1% agarose gel. Bands at 550 bp were quantified.

### Plasma SCFA profiling

Plasma SCFA concentrations were quantified using an Agilent 7890–5977 gas chromatography-mass spectrometry (GC–MS, Agilent, Blacksburg, VA, USA). Plasma samples were collected from old and young mice or 2 weeks after the mice were transplanted with young or old mouse fecal solution, stored at − 80°C and thawed to room temperature for processing. Briefly, 100-μl plasma sample was mixed with 900-μl ethanol (containing 0.5% HCl, V/V) and vortex-mixed. Ultrasonic treatment was applied for 40 min, and then, the samples were centrifuged at 14,000 RPM for 10 min. The supernatant of samples was measured by GC–MS analysis on an Agilent 7890–5977 GC–MS with electron impact ionization and a DB-FFAP capillary column (30 m × 0.25 mm × 0.25 μm).

Valeric acid in the blood harvested from the experiment to determine the dosage of valeric acid injected intraperitoneally was measured by using an ELISA kit (catalog number: JM-12270M1, Jingmei Biotechnology, Jiangsu, China) following the manufacturer’s instructions.

### Serum and brain tissue harvesting

Mice were weighed and anesthetized with isoflurane at 24 h after MCAO. Blood samples were obtained from the right heart after thoracotomy. The blood was centrifuged (13,000 g, 4°C) after it had been placed at 4°C for 2 h to harvest the serum. After the collection of the blood, mice were perfused with 4°C saline for brain tissue harvesting. The cerebral cortex (anteroposterior: − 1.5 to 2.5 mm, mediolateral: 1.0 to 3.0 mm) was dissected out. All dissection procedures were performed on ice.

### ELISA of interleukins in the serum and brain

IL-10, IL-17, IL-1β, and IL-6 in the serum and brain were detected by using ELISA kits (catalog number: M1000B, M1700, MLB00C and D6050, R&D SYSTEM, Inc., Minneapolis, MN, USA) as we described before [8]. The brain tissues were homogenized on ice in the RIPA buffer containing 25 mM Tris–HCl with pH 7.6, 150 mM NaCl, 1% sodium deoxycholate and 0.1% SDS (catalog number: 89901, Thermo Scientific, Rockford, IL, USA) and a protease inhibitor cocktail containing 10 mg/ml aproteinin, 5 mg/ml peptastin, 5 mg/ml leupeptin, and 1-mM phenylmethane sulfonylfluoride (catalog number: SRE0055, Sigma-Aldrich, St. Louis, MO, USA). The supernatant was collected for ELISA detection after being centrifuged for 20 min (13,000 g, 4°C), and the protein concentration was determined by the BCA method. Cytokines in the serum and supernatant of brain samples were detected according to the manufacturer’s instruction. The amount of IL-10, IL-17, IL-1β, and IL-6 in each brain sample was normalized by its protein content.

### Statistical analysis

**Fig. 2:**
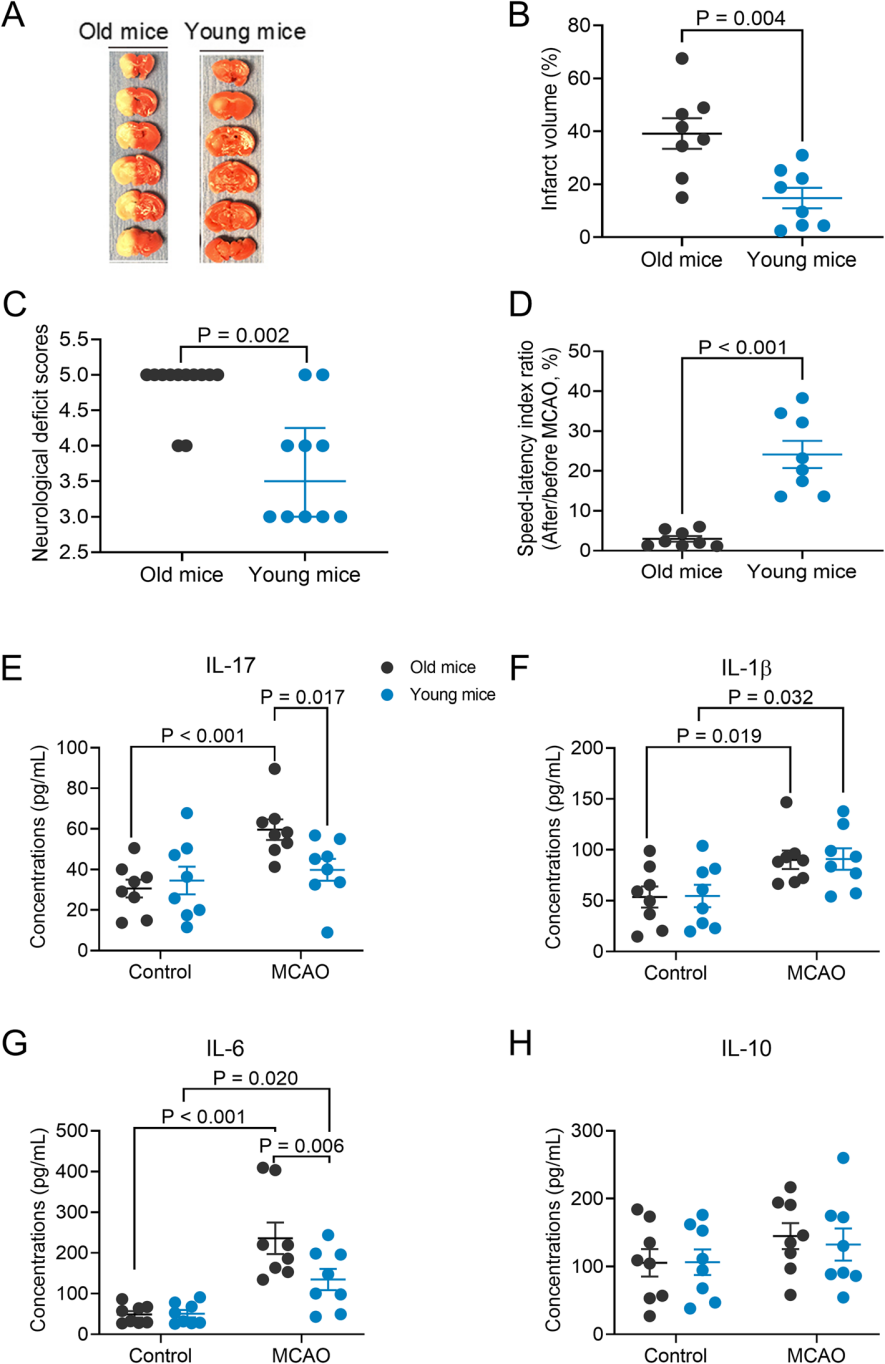
Aging-dependent worsening of neurological outcome and heightened inflammatory response after brain ischemia. Old mice (18 months old) or young mice (8 weeks old) were subjected to 120-min MCAO. Their neurological outcome was evaluated, and blood was collected 24 h after MCAO. **A** representative brain slice images after 2,3,5-triphenyltetrazolium chloride staining, **B** infarct volume, **C** neurological deficit scores, **D** performance on rotarod, **E** IL-17 concentrations in the blood, **F** IL-1β concentrations in the blood, **G** IL-6 concentrations in the blood, and **H** IL-10 concentrations in the blood. Parametric results in normal distribution are in mean ± S.E.M. (panels **B** and **D** to **H**) and other results that are nonparametric data or parametric data in non-normal distribution are presented as median with interquartile range (panel **C**). Data of each individual animal is also presented (*n* = 8–12)

Parametric results in normal distribution are presented as means ± S.E.M. Data are presented as median with interquartile range when they are not in normal distribution or are not parametric. Data of each individual animal is also presented in the figures. Two-way ANOVA was used to determine whether age and brain ischemia were significant factors in determining the cytokine concentrations in the blood (data in Fig. 2E–H). Gut microbiota data were analyzed as described in section “Gut microbiota profiling”. The other data were analyzed by one-way ANOVA followed by Tukey’s test if the data were normally distributed, by one-way ANOVA on rank followed by Tukey test if the data were not normally distributed, by *t* test or rank sum test as appropriate. Body weight data were analyzed by two-way repeated measures ANOVA. Survival curves were analyzed by Mantel-Cox test followed by the Gehan-Breslow-Wilcoxon test. Significant difference was defined as *P* < 0.05. All statistical analyses were performed with SigmaStat (Systat Software, Inc., Point Richmond, CA, USA).

## Results

### Old mice had a worsened neurological outcome and enhanced proinflammatory cytokine production after brain ischemia and changed composition in gut microbiota and increased SCFAs in the blood before brain ischemia

To determine whether aging affected neurological outcome after brain ischemia, 8-week and 18-month-old C57BL/6J male mice were subjected to 120-min left MCAO. They were called young and old mice, respectively, in this study. Old mice had larger infarct brain volumes, higher neurological deficit scores, and worse performance on rotarod than young mice (Fig. 2A–D). These results suggest that old mice have a worse neurological outcome after focal brain ischemia than young mice. Brain ischemia and reperfusion increased IL-1β and IL-6 in the blood of young mice and IL-17, IL-1β, and IL6 in the blood of old mice (Fig. 2E–G). Consistent with the finding of neurological outcome, old mice had higher IL-17 and IL-6 concentrations in their blood after brain ischemia and reperfusion [age was a factor to influence the effects of brain ischemia on IL-17 and IL-6 concentration: *F*(1,28) = 4.673, *P* = 0.039, for IL-17; *F*(1,28) = 4.559, *P* = 0.042, for IL-6], although there was no difference in these cytokines at baseline between young and old mice (Fig. 2E–G). Brain ischemia and reperfusion did not affect the expression of IL-10 (Fig. 2H), an anti-inflammatory cytokine [8], in the blood of old and young mice. These results suggest that brain ischemia induces a heightened inflammatory response in old mice.

**Fig. 3:**
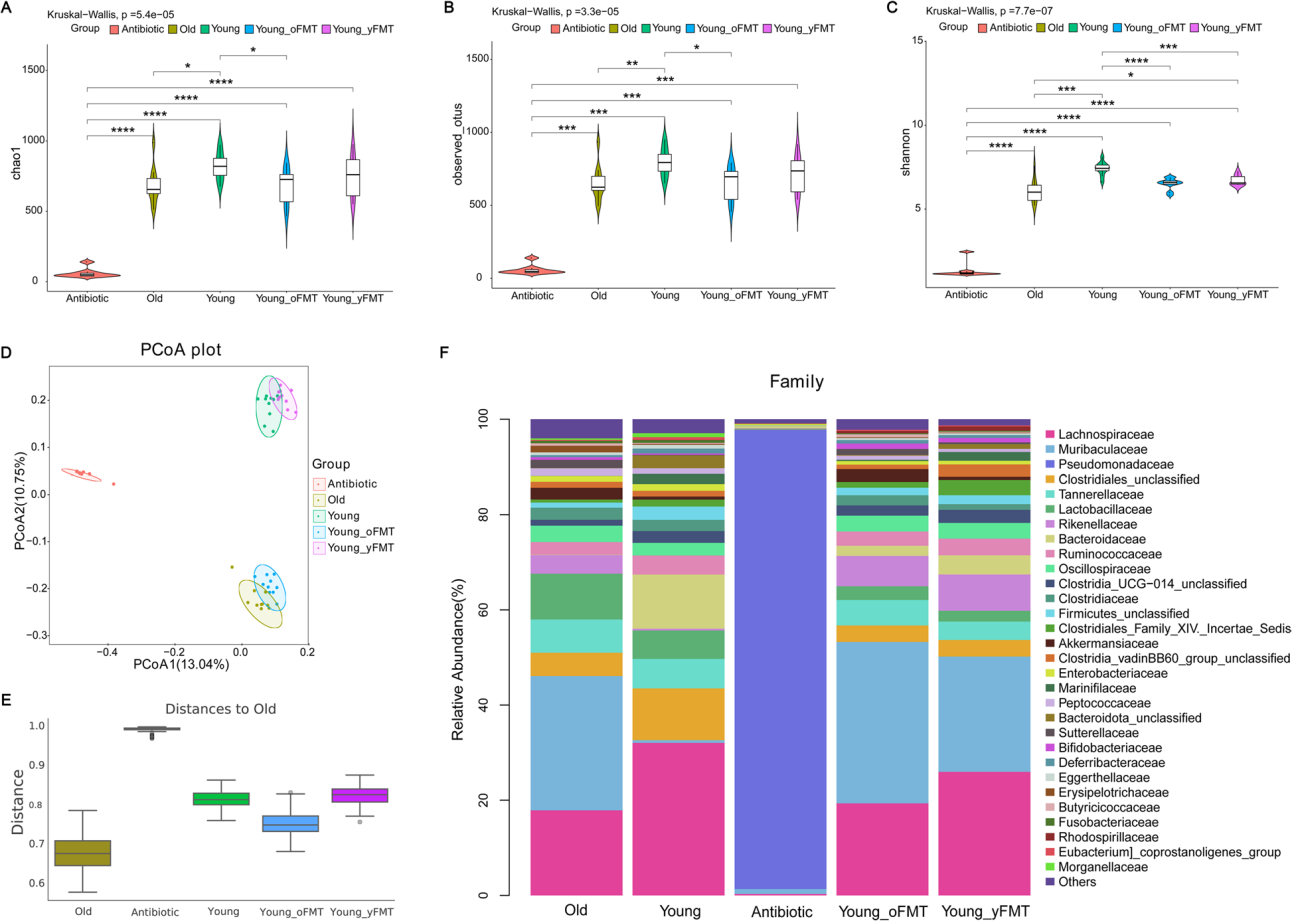
Aging-dependent gut microbiota changes and effects of fecal transplant on gut microbiota. Feces were harvested from old mice (18 months old, *n* = 10), young mice (8 weeks old, *n* = 10), young mice 1 day after the completion of cefazolin treatment (*n* = 8) or young mice 14 days after receiving transplant of feces from young mice (Young-yFMT, *n* = 10) or from old mice (Young-oFMT, *n* = 10). The gut microbiota in these 5 groups of mice was analyzed and compared. **A** to **C** *α* diversity difference among the groups analyzed by chao1, observed_otus, and Shannon methods, respectively. **D**, **E** *β* diversity difference among the 5 groups analyzed by the PCoA and Anosim using Jaccard distance metrics, respectively. *F* stacked bar plot of relative abundance of taxa within gut microbiota at family level. * *P* < 0.05, ** *P* < 0.01, *** *P* < 0.001, and **** *P* < 0.0001

**Fig. 4:**
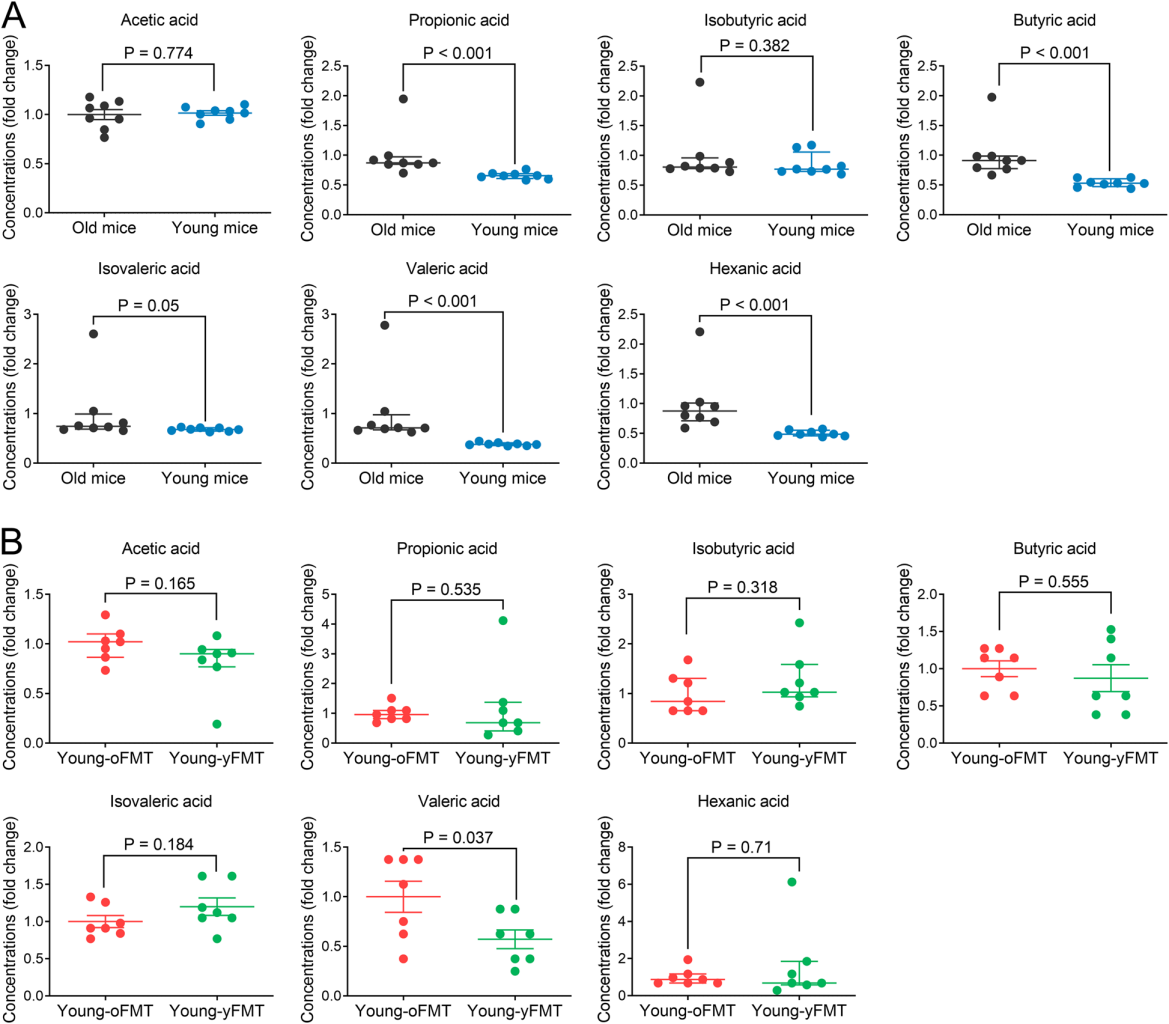
Ageing-dependent changes of short-chain fatty acids in the blood. **A** The blood was collected from old mice (18 months old) or young mice (8 weeks old) without any treatment or brain ischemia and used to measure short-chain fatty acids. **B** The blood was collected from young mice (8 weeks old) transplanted 14 days ago with old mouse (18 months old) (Young-oFMT) or young mouse (8 weeks old) (Young-yFMT) feces. The blood was then used to measure short-chain fatty acids. Parametric results in normal distribution are in mean ± S.E.M. (acetic acid data in panel **A** and butyric acid, isovaleric acid, and valeric acid data in panel **B**) and other results that are nonparametric data or parametric data in non-normal distribution are presented as median with interquartile range (all other panels). Data of each individual animal is also presented (*n* = 8 for panel **A**, = 7 for panel **B**). Results in panels **A** and **B** were normalized by the mean value of the old mice or Young-oFMT groups, respectively

As an initial step to determine the role of gut microbiota in the aging-related changes in ischemic brain injury and inflammation response, gut microbiota was investigated. Gut microbiota in young mice was more diverse than that in old mice as indicated by *α* diversity parameters and had a taxonomic composition different from that of old mice as indicated by *β* diversity parameters (Fig. 3A to E). Young mice had an increased relative abundance of certain bacteria, such as *f_Lachnospiraceae*, *f_Bacteroidaceae*, and *f_Clostridiales_unclassified*. The relative abundance of some bacteria, such as *f_Muribaculaceae* and *f_Lactobacillaceae*, was increased in old mice (Fig. 3F). These results indicate that gut microbiota in the old mice is different from that in the young mice. Since SCFAs are mainly produced from gut microbiota and gut microbiota can affect outcome after brain ischemia [9–11], blood concentrations of SCFAs were measured in mice without brain ischemia. Old mice had an increase of propionic acid, butyric acid, isovaleric acid, valeric acid and hexanic acid (Fig. 4A), suggesting that aging-related changes in gut microbiota composition leads to changes of SCFAs in the host blood.

### Transplanting old mouse feces worsened neurological outcome, heightened proinflammatory cytokine production, and increased valeric acid in the blood of young mice after brain ischemia

To determine whether different gut microbiota plays a role in the worsened neurological outcome after brain ischemia, gut microbiota transplant was performed. These mice were subjected to 120-min left MCAO 2 weeks after fecal transplantation. The gut microbiota was examined prior to MCAO to determine the success of fecal transplant. Similar to the young and old mice without fecal transplantation, Young-oFMT mice had a decreased richness in gut microbiota at 2 weeks after the transplantation compared to young mice, but there was no difference in the richness of gut microbiota between young mice and Young-yFMT mice or between old mice and Young-oFMT mice (Fig. 3A to C). The taxonomic composition of gut microbiota was similar between young mice and Young-yFMT mice or between old mice and Young-oFMT mice (Fig. 3D and E). Specifically, the Young-oFMT mice had an increased relative abundance of *f_Muribaculaceae* and decreased *f_Lachnospiraceae and f_Bacteroidaceae* compared with young mice or Young-yFMT mice (Fig. 3F), a finding that is similar to that of the comparison between young and old mice*.* These results suggest the success of FMT in mice. Of note, cefazolin application very significantly decreased gut microbiota (Fig. 3) [20]. However, the total bacterial load was not different between those mice receiving gut microbiota transplant and those without transplant (Fig. S1), suggesting that the overall gut microbiome load has recovered after cefazoline treatment in the mice with transplant before they were subjected to brain ischemia.

**Fig. 5:**
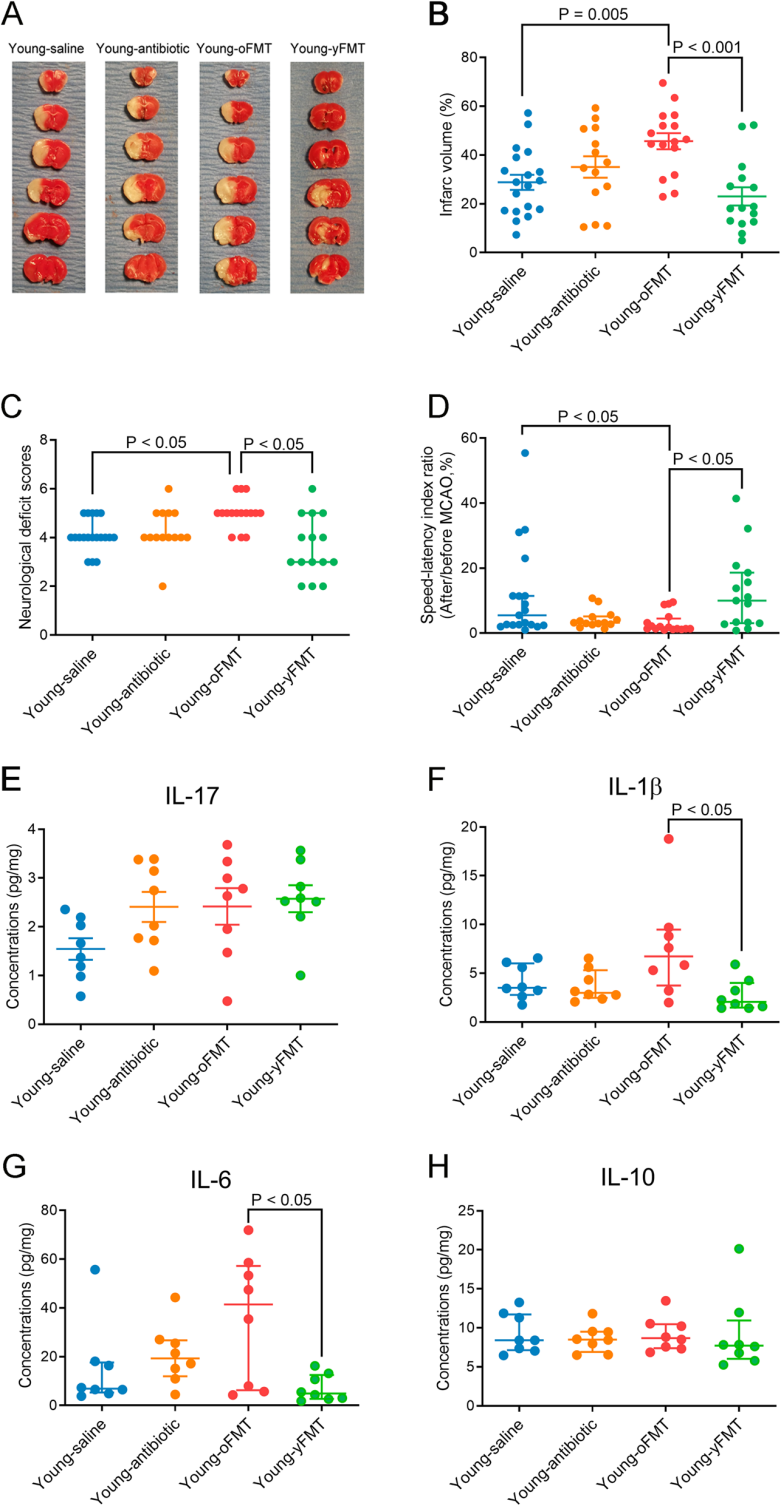
Worsening of neurological outcome and heightened inflammatory responses in the brain after brain ischemia in young mice transplanted with old mouse feces. Young mice (8 weeks old) received saline, cefazolin, cefazolin, and then transplantation of old mouse feces or cefazolin and then transplantation of young mouse feces. They were subjected to 120-min MCAO 2 weeks after the fecal transplantation and named Young-saline, Young-antibiotic, Young-oFMT, and Young-yFMT, respectively. Their neurological outcome was evaluated and brain was collected 24 h after MCAO. **A** Representative brain slice images after 2,3,5-triphenyltetrazolium chloride staining. **B** Infarct volume. **C** Neurological deficit scores. **D** Performance on rotarod. **E** IL-17 concentrations in the brain. **F** IL-1β concentrations in the brain. **G** IL-6 concentrations in the brain. **H** IL-10 concentrations in the brain. One cohort of mice was used to generate the data presented in panels **A** to **D**. Another cohort was used for the data presented in panels **E** to **H**. Parametric results in normal distribution are in mean ± S.E.M. (panels **B** and **E**) and other results that are nonparametric data or parametric data in non-normal distribution are presented as median with interquartile range (all other panels). Data of each individual animal is also presented (*n* = 14–19 for panels **B** to **D**, = 8 for panels **E** to **H**)

Young-oFMT mice had larger brain infarct volumes, higher neurological deficit scores and worse performance on rotarod than young mice without fecal transplantation or Young-yFMT mice (Fig. 5A–D). These results suggest that old mouse microbiota may contribute to the worse neurological outcome after brain ischemia. Young-oFMT mice had higher IL-1β and IL-6 concentrations in the left frontal cerebral cortex area 1 (Fr1), an ischemic penumbral region [8, 28]. Transplantation of young or old mouse feces did not change the levels of IL-17 or IL-10 in the Fr1 (Fig. 5E-H). These results suggest that young mice with transplantation of old mouse feces had a heightened inflammatory response, a condition similar to that of old mice. However, the body weights of mice in the four groups before brain ischemia were not different (Fig. S2A-C), suggesting that the general conditions of these mice with fecal transplantation were similar to those without fecal transplantation. Antibiotic treatment that was necessary before fecal transplant did not affect the weight increase curve (Fig. S2D). Also, the magnitude of cerebral blood flow decrease during MCAO was similar between mice transplanted with young mouse or old mouse feces (Fig. S2E). These results suggest that the worsened neurological outcome of mice transplanted with old mouse feces is not due to different levels of cerebral blood flow decrease or poor general condition.

**Fig. 6:**
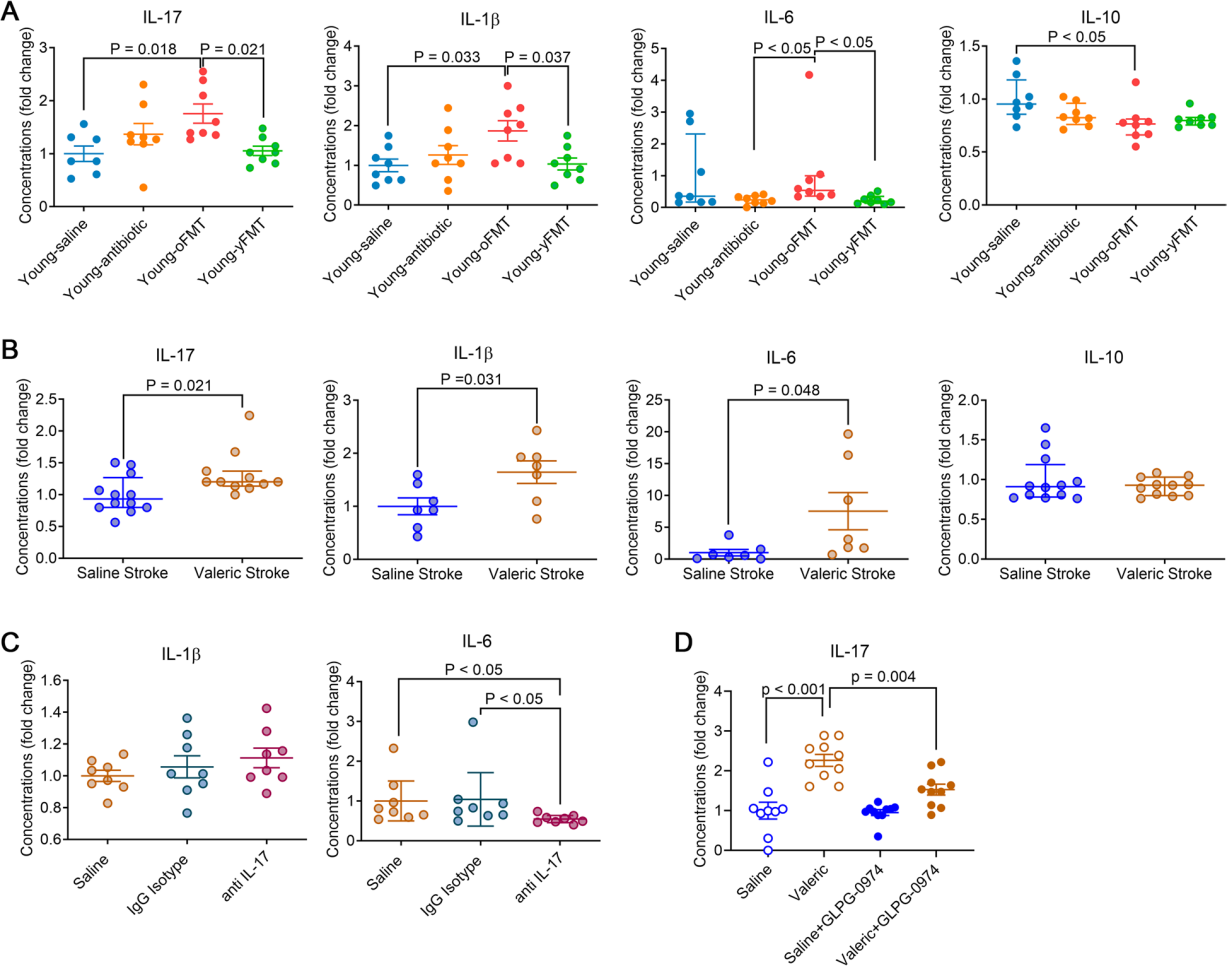
Heightened inflammatory responses in the blood after brain ischemia in young mice transplanted with old mouse feces or treated with valeric acid. The blood was harvested 24 h after 120-min MCAO for cytokine analysis. **A** Young mice (8 weeks old) received saline, cefazolin, cefazolin, and then transplantation of old mouse feces or cefazolin and then transplantation of young mouse feces. They were subjected to 120-min MCAO 2 weeks after the fecal transplantation and named Young-saline, Young-antibiotic, Yyoung-oFMT, and Young-yFMT, respectively. **B** Young mice received saline or valeric acid intraperitoneally and then subjected to 120-min MCAO. They were named saline stroke and valeric stroke, respectively. **C** Young mice received valeric acid and were subjected to 120-min MCAO. They were then received intravenous saline, mouse IgG1, or mouse monoclonal anti-IL-17 antibody and named saline, IgG isotype, and anti-IL-17, respectively. **D** Young mice received intraperitoneal normal saline, valeric acid, the combination of saline and GLPG-0974 or the combination of valeric acid and GLPG-0974. IL-17 in the blood was measured. Parametric results in normal distribution are in mean ± S.E.M. (IL-17 and IL-1β data in panel **A**, IL-1β and IL-6 data in panel **B**, and IL-1β data in panel **C**) and other results that are nonparametric data or parametric data in non-normal distribution are presented as median with interquartile range (all other panels). Data of each individual animal is also presented (*n* = 8 for panel **A**, = 7–11 for panel **B**, = 8 for panel **C**, *n* = 9–10 for panel **D**). Results in panels **A** to **D** were normalized by the mean value of the Young-saline, saline stroke, saline, and saline groups, respectively

Similar to the inflammatory cytokine changes in the ischemic penumbral region, Young-oFMT mice had increased IL-17, IL-1β, and IL-6 in the blood compared with young mice without fecal transplantation or Young-yFMT mice. In addition, Young-oFMT mice had decreased IL-10 compared to young mice without fecal transplantation (Fig. 6A). These results suggest that brain ischemia and reperfusion induced a heightened inflammatory response in Young-oFMT mice.

Since there was a significant difference in the neurological outcome and inflammatory response in the blood and ischemic penumbral brain tissues between young mice transplanted with young and old mouse feces, we determined whether there was a difference in SCFAs between these mice. Young-oFMT mice had increased valeric acid in the blood compared with Young-yFMT mice. The other SCFAs were not different between these two types of mice (Fig. 4B). These results suggest a potential role of valeric acid in mediating the effects of old mouse gut microbiota. Interestingly, the colon mucosal permeability of Young-oFMT mice was higher than that of Young-yFMT mice. MCAO increased this permeability in those two types of mice, but the colon mucosal permeability remained higher in the Young-oFMT mice with MCAO (Fig. S2F). These results suggest that gut microbiota of old mice may increase colon mucosal permeability and, therefore, may facilitate the absorption of toxic agents from colon into the circulation.

### Valeric acid worsened neurological outcome and heightened proinflammatory cytokine production in young mice after brain ischemia

**Fig. 7:**
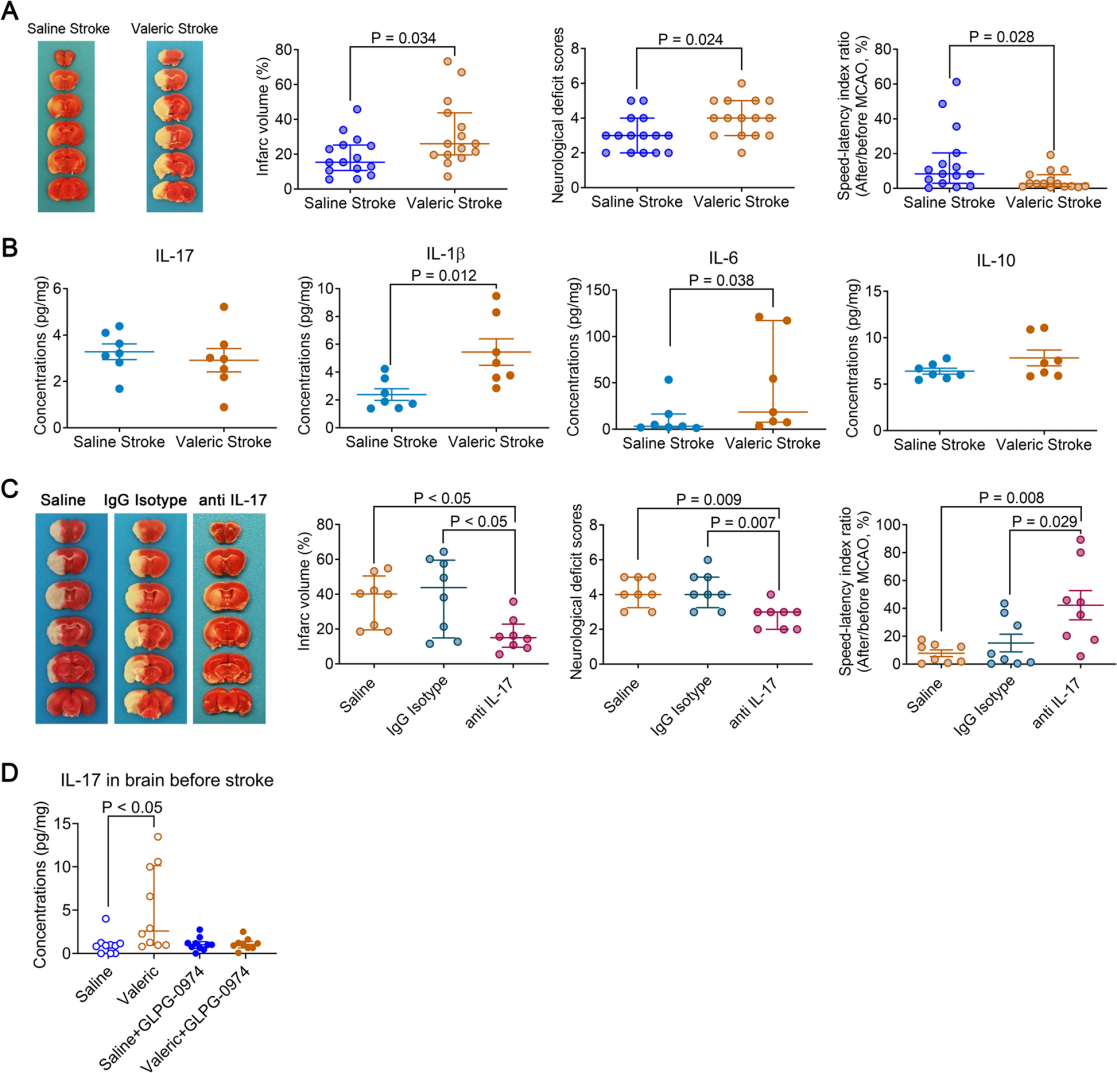
Worsening of neurological outcome and heightened inflammatory responses in the brain after brain ischemia in young mice treated with valeric acid and improved outcome by an anti-IL-17 antibody. Young mice (8 weeks old) received intraperitoneal saline or valeric sodium. They were subjected to 120-min MCAO and named saline stroke and valeric stroke, respectively. Their neurological outcome was evaluated, and the brain was collected 24 h after MCAO for cytokine measurements (results are in panels **A** and **B**). In another experiment, young mice (8 weeks old) were subjected to 120-min MCAO plus intraperitoneal valeric acid and then received intravenous saline, mouse IgG1, or mouse monoclonal anti-IL-17 antibody. They were named saline, IgG isotype, and anti-IL-17, respectively. Their neurological outcome was evaluated (results are in panel **C**). **A** Neurological outcome. B Inflammatory markers in the brain. **C** Neurological outcome. **D** Young mice received intraperitoneal normal saline, valeric acid, the combination of saline and GLPG-0974 or the combination of valeric acid and GLPG-0974. The cerebral cortex was harvested for measuring IL-17. Parametric results in normal distribution are in mean ± S.E.M. (IL-17, IL-1β, and IL-10 data in panel **B**) and other results that are nonparametric data or parametric data in non-normal distribution are presented as median with interquartile range (all other panels). Data of each individual animal is also presented (*n* = 15 for panel **A**, = 7 for panels **B**, = 8 for panel **C**, *n* = 9–10 for panel **D**)

To determine the role of valeric acid in brain ischemic injury, young mice received 100 mg/kg valeric sodium intraperitoneally at 1 h before and 6 h after the MCAO. Our pilot study showed that this regimen increased the concentrations of valeric acid in the blood of young mice to a level similar to that in the old mice (Fig. S3). Valeric acid increased the concentrations of IL-17, IL-1β, and IL-6 in the blood of mice with experimental stroke (Fig. 6B). These results suggest that valeric acid enhances the inflammatory response after stroke. Young mice that received valeric sodium had a larger infarct volume, worse neurological deficit scores, and a poorer performance on rotarod than young mice that received saline injection (Fig. 7A). Valeric sodium also increased IL-1β and IL-6 in the ischemic penumbral brain tissues (Fig. 7B). These results suggest that valeric acid may enhance inflammatory response and increase brain ischemic injury.

### Blocking IL-17 in the blood attenuated valeric acid-induced worsening of neurological outcome and heightening of proinflammatory cytokine increase

IL-17 can regulate the production of chemokines and other proinflammatory cytokines, such as IL-1β and IL-6 [17, 18]. Our results described above showed that transplantation of old mouse feces or intraperitoneal injection of valeric acid did not increase IL-17 in the brain but increased IL-17 in the blood of mice with brain ischemia (Figs. 5E, 6A, B, and 7B). On the other hand, IL-1β and IL-6 were increased in the blood and brain in stroke mice that received valeric acid or transplantation of old mouse feces (Figs. 5F, G, 6A, B, and 7B). Thus, IL-17 in the blood could be a molecule that can easily be targeted without the concern of additional production sites, such as brain, in the case of IL-1β and IL-6.

To determine the role of IL-17 in valeric acid effects on brain ischemic injury, young mice that received intraperitoneal injection of valeric sodium as described above had an intravenous injection of 200 μl saline, 100-μg mouse IgG1 in 200 μl, or 100-μg mouse monoclonal anti-IL-17 antibody in 200 μl 2 h after the initiation of MCAO. The antibody but not the mouse IgG1 reduced IL-6 concentrations in the blood (Fig. 6C). The anti-IL-17 antibody but not the mouse IgG1 reduced brain infarct volume and improved neurological function of mice that received valeric acid (Fig. 7C). These results suggest that IL-17 neutralization reduced valeric acid-induced worsening of brain ischemic injury and inflammatory response.

As presented above, IL-17 appeared to be a molecule downstream of valeric acid to mediate the worsened neurologic outcome with aging after brain ischemia. Four FFARs have been identified. FFAR1 and FFAR4 are for long chain fatty acids. FFAR2 and FFAR3 are for SCFAs. FFAR2 are expressed in adipocytes, enteroendocrine cells, immune cells, and white blood cells. FFAR3 are expressed in adipocytes, enteroendocrine cells, and peripheral nervous tissues [29]. Thus, GLPG-0974, a FFAR2 antagonist [19], was used. GLPG-0974 blocked the increase of IL-17 in the blood and brain caused by valeric acid in mice without MCAO (Figs. 6D and 7D). These results suggest that valeric acid activates FFAR2 to increase IL-17.

### Transplanting with old mouse feces worsened general outcome of young mice and transplanting with young mouse feces improved general outcome of old mice

**Fig. 8:**
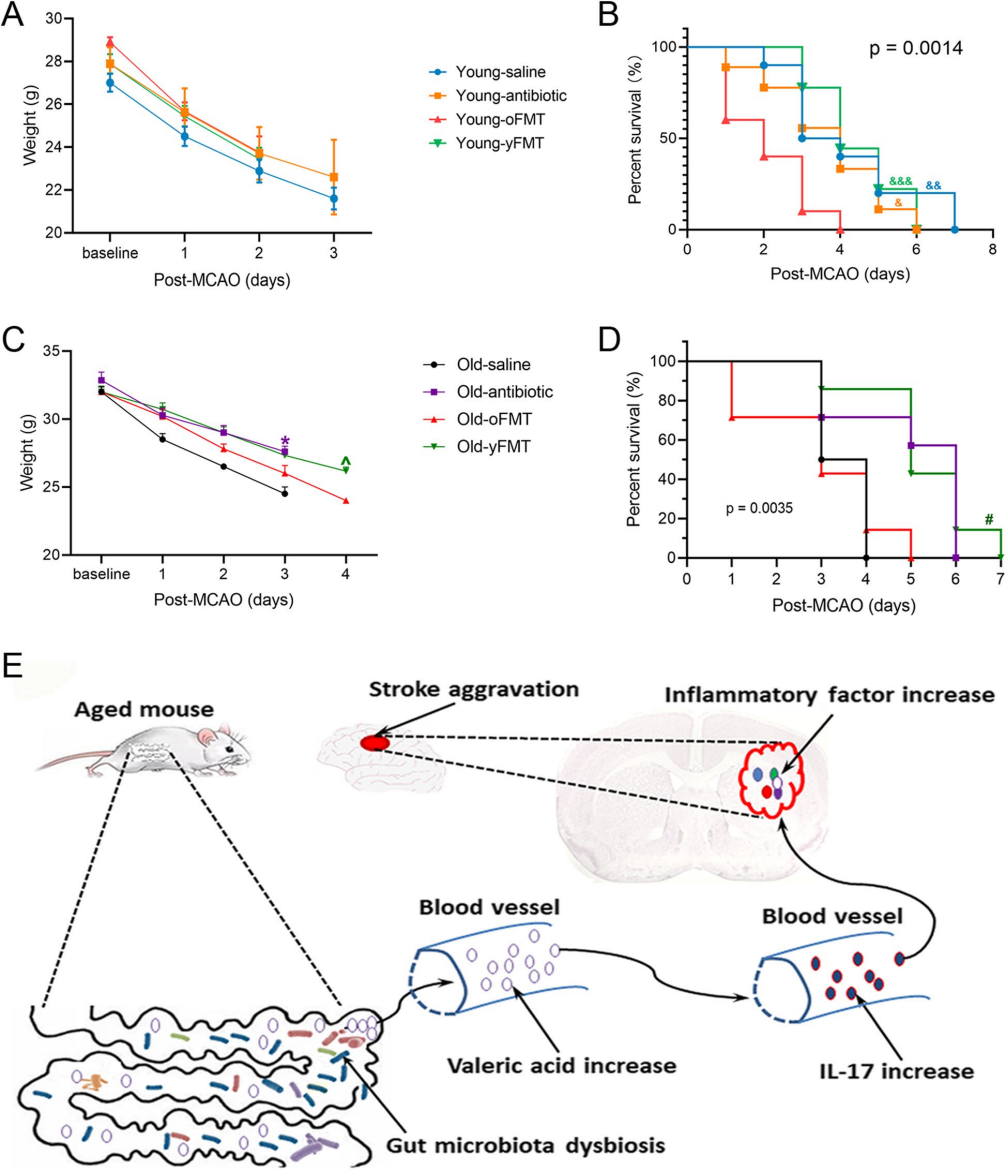
Worsened outcome in young mice transplanted with old mouse feces and improved outcome in old mice transplanted with young mouse feces. Young mice (8 weeks old) received saline, cefazolin, cefazolin, and then transplantation of old mouse feces or cefazolin and then transplantation of young mouse feces. They were subjected to 120-min MCAO 2 weeks after the fecal transplantation and named Young-saline, Young-antibiotic, Young-oFMT, and Young-yFMT, respectively. In another experiment, old mice (18 months old) received saline, cefazolin, cefazolin, and then transplantation of old mouse feces or cefazolin and then transplantation of young mouse feces. They were subjected to 60-min MCAO 2 weeks after the fecal transplantation and named Old-saline, Old-antibiotic, Old-oFMT, and Old-yFMT, respectively. Their body weights and survival curves were evaluated after MCAO. **A** Body weight changes of young mice. **B** Survival curve of young mice. **C** Body weight changes of old mice. **D** Survival curve of old mice. **E** Summary diagram for the mechanism of aging-dependent microbiota changes-induced increase of ischemic brain injury and inflammatory responses. Results in panels **A** and **C** are in mean ± S.E.M. (*n* = 4 to 9 for panel **A** and = 6–7 for panel **C**). *F(1, 11) = 8.635, *P* = 0.013 compared with old-saline mice. ^*F*(1,12) = 5.383, *P* = 0.034 compared with Old-oFMT mice. The survival curves of the four groups in panels **B** and **D** were different when analyzed by the Mantel-Cox test (*P* = 0.0014 for panel **B** and = 0.0035 for panel **D**). ^&^*P* < 0.05, ^&&^*P* < 0.01, ^&&&^*P* < 0.001 compared with Young-oFMT mice by the Gehan-Breslow-Wilcoxon test; ^#^*P* < 0.05 compared with Old-oFMT or Old-saline by the Gehan-Breslow-Wilcoxon test

To provide additional evidence for the role of gut microbiota in determining the outcome after brain ischemia, body weights and mortality were recorded after brain ischemia. Young mice lost body weights after MCAO whether they had fecal transplant (Fig. 8A). Young-oFMT mice had a worse survival curve than control young mice, young mice receiving antibiotic treatment, or Young-yFMT mice (Fig. 8B). These results suggest that transplanting young mice with old mouse feces worsened the outcome of young mice after brain ischemia. In another experiment, Old-yFMT or Old-oFMT mice were subjected to a 60-min left MCAO. Old-yFMT mice had less body weight loss over time after the MCAO than Old-oFMT mice [*F*(1,12] = 5.383, *P* = 0.034] (Fig. 8C). In addition, Old-yFMT mice had a better survival curve than Old-oFMT mice or old mice without fecal transplantation (Fig. 8D). These results suggest that transplantation with young mouse feces improves the outcome of old mice after brain ischemia.

## Discussion

Aging is a strong independent risk factor for ischemic stroke in humans [1, 2]. Elderly patients with ischemic stroke also have a worse outcome including higher mortality and morbidity rates and poorer neurological function recovery than young patients [3, 4]. Similar to this clinical situation, old mice had a worse neurological outcome than young mice in our study. Old mice also had an exaggerated inflammatory response to ischemic stroke, one possible mechanism for the worsened neurological outcome. Since gut microbiota is known to regulate immune maturation and inflammatory response [9, 30], we hypothesize that gut microbiota contributes to this different inflammatory response. Our results clearly showed that the gut microbiota in young mice is more diverse than that in old mice. Old mice had a relatively abundant amount of *f_Muribaculaceae* and *f_Lactobacillaceae*. Gut microbiota in young mice was rich in *f_Lachnospiraceae*, *f_Bacteroidaceae*, and *f_Clostridiales_unclassified*. *f_Bacteroidaceae* is in the phylum Bacteroidetes. While *f_Muribaculaceae* is in the phylum Bacteroidota, *f_Lactobacillaceae* is in the phylum Firmicutes. Increased Firmicutes is considered a marker of aging-related changes in gut microbiota [11]. Our results suggest that old mice have gut dysbiosis.

To determine whether aging-related changes in gut microbiota contributes to the worsened neurological outcome in old mice, we used cefazolin to eliminate the existing gut microbiota of young mice as shown in our pervious study [20]. These mice were then transplanted with old or young mouse feces. This transplant changed the gut microbiota of recipients to the microbiota that was similar to that of donors. In addition, Young-oFMT mice had an increased valeric acid concentration in the blood compared with Young-yFMT mice, a similar finding when comparing old mice with young mice. These results suggest that transplantation renders young mice to have gut microbiota similar to that transplanted. Young-oFMT mice had a worsened neurological outcome and heightened inflammatory response in the blood and ischemic penumbral brain tissues, a situation similar to that of old mice. Young-oFMT mice also had a higher mortality rate than Young-yFMT mice. Consistent with our findings, a previous study showed that transplantation of old mouse feces worsened the survival rates and neurological functions of the young recipients [11]. In addition, our current study showed that Old-yFMT mice lost less body weight and survived better than Old-oFMT mice. These results suggest that a difference in gut microbiota contributes to the worsened ischemic brain injury, inflammatory response, and general outcome after brain ischemia in old mice compared with young mice.

Gut microbiota produces various metabolites. Among them, SCFAs in humans are mostly produced by gut microbiota [15]. SCFAs have been shown to regulate immune response [13, 14, 31]. To determine whether SCFAs mediate the effects of gut microbiota on the outcome of ischemic stroke, blood levels of SCFAs, and gut mucosal permeability were measured. Gut mucosal permeability was higher in old mice than that in young mice no matter whether the mice had MCAO. These results suggest that mucosa in the old mice may let more chemicals including SCFAs get into the circulation. This situation was worsened when mice had ischemic stroke, similar to a finding reported previously [32]. Old mice had an increased blood level of 5 SCFAs among the 7 SCFAs measured in the study. However, valeric acid was the only one that was increased in the mice transplanted with old mouse feces. Since these mice also had a worse neurological outcome and heightened inflammatory response in the blood and brain tissues, these results suggest that valeric acid in the blood may be a mediator for these effects of old mouse gut microbiota. In supporting this idea, intraperitoneal valeric acid worsened neurological outcome and heightened the inflammatory response in the blood and ischemic penumbral brain tissues. Thus, our results strongly suggest that valeric acid mediates the effects of old mouse gut microbiota on neurological outcome and inflammatory response.

To determine a mediator downstream of valeric acid, we used an IL-17 neutralizing antibody. IL-17 in the blood was increased by intraperitoneal valeric acid. IL-17 in the ischemic brain tissues was not affected by valeric acid. Thus, neutralizing IL-17 in the blood could have an effect if IL-17 is a downstream mediator for valeric acid. The mouse monoclonal anti-IL-17 antibody improved neurological outcome and reduced inflammation response of young mice after the combination of ischemic stroke and valeric acid. IL-17 in the blood of young mice after ischemic stroke was not changed compared to young mice without ischemic stroke, suggesting that IL-17 is a downstream mediator for old mouse gut microbiota- and valeric acid-induced worsening of neurological outcome and increase of inflammatory response. Since the increase of IL-17 caused by valeric acid was inhibited by an FFAR2 inhibitor, valeric acid-induced increase of IL-17 may be mediated by FFAR2. Consistent with this novel finding, our previous study has shown that valeric acid induces immune and inflammatory responses [22] and FFAR2 mediates *fusobacterium nucleatum*-induced IL-17 production in the colon [33]. Thus, the novel pathway, gut microbiota-valeric acid-FFAR2-IL-17, may be an underlying mechanism for the worsened neurological outcome and heightened inflammatory response in old mice (Fig. 8E).

Previous studies have shown that aging is associated with decreased SCFAs [11, 15]. However, those studies measured SCFAs in the feces. Our results clearly showed that SCFAs were increased in the blood of old mice. Old mice had an increase in colon mucosal permeability, which may facilitate the absorption of SCFAs. Thus, part of the decrease of SCFAs in feces with aging as shown in the previous studies may be due to the absorption of SCFAs into the blood. In addition, a previous study showed that the neurological outcome in rats was improved with increase of acetic acid, butyric acid, and valeric acid [34]. However, the measurement of these SCFAs was also on feces. These results suggest the importance to measure SCFAs in the blood to know the possible role of SCFAs in ischemic brain injury. A human study has shown that stroke patients have increased valeric acid in their feces and this increase was associated with increased white blood cells and high sensitive C-reactive protein in the blood [16]. However, the role of this increased valeric acid in feces in stroke and inflammatory response was not investigated. Studies have shown that supplementing the combination of butyrate, acetate, and propionate improve neurological outcome after brain ischemia [13]. Treating rats with brain ischemia by butyric acid alone also improve neurological outcome. Butyrate can inhibit proinflammatory cytokine production [34]. Our results suggest a detrimental effect of valeric acid in ischemic stroke. Thus, different SCFAs may have different effects on neurological outcome after brain ischemia. In addition to modulating immune function, other functions, such as restoring intestinal homeostasis [31], may also be a mechanism for their effects on ischemic brain injury.

The role of gut microbiota in ischemic brain injury has been shown. Treatment with broad-spectrum antibiotics worsened neurological outcome in mice [35]. Replacing germ-free mice with normal gut microbiota improved neurological outcome [10]. These results suggest a role of normal gut microbiota in protecting mice against ischemic brain injury. However, another study showed that antibiotics-induced gut dysbiosis was protective. The effects may be due to the reduction of IL-17 + γδ T cells and increase of regulatory T cells [9]. Although the outcome seems contradictory among these studies, these three studies strongly suggest a role of gut microbiota in determining the degree of ischemic brain injury. Additional evidence for supporting this role came from a recent study showing that transplanting with old mouse feces worsened the functional outcome but did not affect the infarct brain volumes after ischemic stroke [11]. Our study has added evidence suggesting that the aging-related worsening of neurological functional and structural outcome, heightened inflammatory response in the brain and poor general outcome may be due to aging-related gut microbiota changes.

Our results suggest that IL-17 is a mediator downstream of valeric acid for the worsened neurological outcome with aging. Consistent with our findings, decreased IL-17 is considered a mechanism for the improved neurological outcome induced by altered gut microbiota after antibiotic treatment [9]. Intravenous use of an anti-IL-17 antibody has been shown to protect against ischemic brain injury in mice [36]. Possible mechanisms for circulating IL-17 to induce brain injury include enhancing oxidative stress and causing injury to the blood–brain barrier [37]. Our results showed that IL-1β and IL-6 were increased in the blood of mice with brain ischemia and that valeric acid enhanced this increase. Neutralizing IL-1β by an intravenous anti-IL-1β antibody improves neurological outcome after stroke in animals [38, 39]. IL-6 in the blood has been considered a biomarker for poorer neurological outcome including larger infarct volume in humans [40]. Thus, the increased IL-1β and IL-6 in the blood may contribute to the worsened neurological outcome with aging after brain ischemia.

Our results of gut microbiota profiling indicate the success of gut microbiota transplantation. In supporting this success, Young-oFMT mice had a worse neurological outcome and neuroinflammatory response after brain ischemia than Young-yFMT mice, which is similar to the situation for the comparison between old mice and young mice after brain ischemia. These results suggest a critical role of gut microbiota changes with aging in the worsened neurological outcome in old mice.

Our study has significant implications. We identified the detrimental role of valeric acid in the experimental ischemic stroke, suggesting that not all SCFAs are beneficial and that it is necessary to measure individual SCFA. A previous study showed a trend of decrease in valeric acid of aged mouse feces [11]. Also, studies have shown a fecal decrease of SCFAs with aging [11, 15]. Our study has illustrated that it is necessary to measure the concentrations of SCFAs in the blood to fully know the effects of SCFAs. More importantly, our results suggest that valeric acid and IL-17 are possible targets for improving neurological outcome after ischemic stroke. We have not found a specific antagonist for valeric acid. However, *Megasphaera massiliensis* is known to produce valeric acid [41]. Methods to inhibit the production of valeric acid from this bacterium or other bacteria may be identified to reduce valeric acid in the blood. Fecal transplantation with healthy gut microbiota may be a way to decrease *Megasphaera massiliensis* and to reduce valeric acid. Changing diet may be another method to alter the composition of gut microbiota [42]. We also showed the importance of IL-17 in aging-related worsening of ischemic brain injury. IL-17 in the blood can be neutralized by its antibody as shown in this study. Several clinical studies are evaluating the treatment potential of monoclonal anti-IL-17 antibody in inflammatory diseases/conditions and its pharmacokinetic and safety profiles (https://clinicaltrials.gov/). This antibody could become an agent to treat ischemic brain injury. Finally, our study suggests a role of FFAR2 in mediating the increase of IL-17 caused by valeric acid. Inhibiting FFAR2 may be an intervention for improving the outcome after brain ischemia in old population.

This study has limitations. Our study was performed in mice. The diet and many other factors that can change gut microbiota are well controlled in them. Also, mouse gut microbiota is different from that of humans. Thus, one cannot extrapolate our results directly to humans. Nevertheless, increased expression of IL-17 mRNA in the blood cells of patients with ischemic stroke has been reported [43]. Also, we used male mice in our study. Additional studies are needed to determine the role of the gut microbiota-valeric acid-FFAR2-IL-17 pathway in ischemic brain injury in female animals. Finally, FMT requires the use of antibiotic(s) to eliminate the gut microbiota of the recipients to facilitate the establishment of the transplanted microbiota in the recipient. Cefazolin was used for this purpose. Cefazolin has direct anti-inflammatory effects and affects learning and memory possibly due to gut dysbiosis in mice [20]. These effects may confound our findings in this study. However, cefazolin treatment was completed 15 days before the MCAO was applied to our animals. Gut microbiota recovered very well at this time after FMT. Cefazolin alone did not affect the neurological outcome after MCAO in this study. Thus, the confounding effects of cefazolin on the findings of this study are limited.

## Conclusions

We have shown that old male mice have a worse neurological outcome and higher inflammatory response after experimental ischemic stroke than young male mice. These effects may be at least partly mediated by the gut microbiota-valeric acid-FFAR2-IL-17 pathway. Interventions on this pathway may hold potential to improve neurological outcome after ischemic stroke.

### Supplementary Information

**Additional file 1:** **Tables S1.** Key resources table.

**Additional file 2:** **Fig. S1.** Effects of gut microbiota transplant on the total bacterial load in the feces. Young mice (8-weeks old) received transplantation of young mouse (8 – 10 weeks old) feces (Young-yFMT) or old mouse (18–21 months old) feces (Young-oFMT). Feces were harvested 14 days after the transplantation and the gut microbiota was analyzed. Top panel: a representative image of 16S rRNA PCR products. Bottom panel: quantification of the products. Results are in mean ± S.E.M. (*n* = 8). **Fig. S2.** Body weight, cerebral blood flow decrease during middle cerebral artery occlusion and colon mucosal permeability of mice with or without fecal transplantation. Young mice (8-weeks old) received saline, cefazolin, cefazolin and then transplantation of old mouse feces or cefazolin and then transplantation of young mouse feces. They were subjected to 120-min MCAO 2 weeks after the fecal transplantation and named Young-saline, Young-antibiotic, Young-oFMT and Young-yFMT, respectively. Young mice transplanted with old or young mouse feces but without MCAO were called Young-oFMT-Sham and Young-yFMT-Sham, respectively. A: body weights at baseline. B: body weights after the treatment of cefazolin. C: body weights 14 days after fecal transplantation. D: body weights of control mice and mice treated with cefazoline during the period from before to 21 days after the first dose of cefazolin treatment. E: cerebral blood flow (CBF) changes. F: colon mucosal permeability. Parametric results in normal distribution are in mean ± S.E.M. (panels C, D and F) and other results that are nonparametric data or parametric data in non-normal distribution are presented as median with interquartile range (all other panels). Data of each individual animal is also presented (*n* = 15 for panels A to C, *n* = 9 – 12 for panel D, *n* = 8 – 12 for panel E). **Fig. S3.** Determination of the dosage of valeric sodium used in the study. Young mice received two intraperitoneal 100 mg/kg valeric sodium in 100 μl or 100 μl saline at an interval of 7 h. Old mice receiving saline injection were also included. Their blood was harvested 3 h after the second injection for measuring valeric acid. Results are in mean ± S.E.M. Data of each individual animal is also presented (*n* = 8).

## Data Availability

The obtained 16S rRNA gene sequences for this study have been deposited in the ArrayExpress database at EMBL-EBI (www.ebi.ac.uk/arrayexpress) under the accession number E-MTAB-13206. Additional information for 16S rRNA sequences used in this study has been deposited in Figshare (https://doi.org/10.6084/m9.figshare.23710527.v2).

## Abbreviations

Old-oFMT: Old mice receiving transplant of feces from old mice
Old-yFMT: Old mice receiving transplant of feces from young mice
FFAR: Free fatty acid receptor
IL: Interleukin
MCAO: Middle cerebral artery occlusion
PBS: Phosphate-buffered saline
SCFA: Short-chain fatty acid
Young-oFMT: Young mice receiving transplant of feces from old mice
Young-yFMT: Young mice receiving transplant of feces from young mice
