# Supplementary material for: Gut microbiota of old mice worsens neurological outcome after brain ischemia via increased valeric acid and IL-17 in the blood
Source: Microbiome. 2023 Sep 12;11:204. doi: 10.1186/s40168-023-01648-1 (PMC10496352; doi:10.1186/s40168-023-01648-1)
Supplement: Supplementary file 2 — Additional file 1: Tables S1. Key resources table. [file 40168_2023_1648_MOESM1_ESM.docx]

**KEY RESOURCES TABLE**

| **REAGENT or RESOURCE** | **RESOURCE** | **IDENTIFIER** |
| --- | --- | --- |
| **Antibodies** |  |  |
| IgG1 kappa isotype | Thermo Scientific | Cat#14-4714-85 |
| mouse IL-17A monoclonal antibody | Thermo Scientific | Cat#16-7173-85 |
| GLPG-0974 | Sigma-Aldrich | Cat#SML2443 |
| **Drugs** |  |  |
| Valeric sodium | Toronto Research Chemicals | Cat#V091420 |
| **Critical Commercial Assays** |  |  |
| Nylon cell strainer | Fisher Scientific | Cat#08-771-2 |
| Monofilament nylon suture | Beijing CiNontech Co. Ltd. | Cat#1622-A1 |
| DNA isolation kit | QIAGEN | Cat#12855-100 |
| AMPure XP magnetic beads | Beckman Coulter | Cat#A63881 |
| Nextera XT Index Kit | Illumina | Cat#FC-131-2001 |
| Qubit dsDNA HS assay kit | Thermo Scientific | Cat#Q33230 |
| Mouse IL-10 ELISA kit | R&D SYSTEM | Cat#M1000B |
| Mouse IL-17 ELISA kit | R&D SYSTEM | Cat#M1700 |
| Mouse IL-1β ELISA kit  Mouse IL-6 ELISA kit | R&D SYSTEM  R&D SYSTEM | Cat#MLB00C  Cat# D6050 |
| Mouse valeric acid ELISA Kit | Jingmei Biotechnology | JM-12270M1 |
| **Chemicals** |  |  |
| Evans blue | Sigma-Aldrich | Cat#E2129-10G |
| N-acetylcysteine | Sigma-Aldrich | Cat#PHR1098-1G |
| Formamide | Sigma-Aldrich | Cat#F9037-100 |
| 10×RIPA buffer | Thermo Scientific | Cat#89901 |
| Protease inhibitor cocktail | Sigma-Aldrich | Cat#SRE0055 |
