# Supplementary material for: Gut microbiota of old mice worsens neurological outcome after brain ischemia via increased valeric acid and IL-17 in the blood
Source: Microbiome. 2023 Sep 12;11:204. doi: 10.1186/s40168-023-01648-1 (PMC10496352; doi:10.1186/s40168-023-01648-1)
Supplement: Supplementary file 3 — Additional file 2: Fig. S1. Effects of gut microbiota transplant on the total bacterial load in the feces. Young mice (8-weeks old) received transplantation of young mouse (8 – 10 weeks old) feces (Young-yFMT) or old mouse (18–21 months old) feces (Young-oFMT). Feces were harvested 14 days after the transplantation and the gut microbiota was analyzed. Top panel: a representative image of 16S rRNA PCR products. Bottom panel: quantification of the products. Results are in mean ± S.E.M. (n = 8). Fig. S2. Body weight, cerebral blood flow decrease during middle cerebral artery occlusion and colon mucosal permeability of mice with or without fecal transplantation. Young mice (8-weeks old) received saline, cefazolin, cefazolin and then transplantation of old mouse feces or cefazolin and then transplantation of young mouse feces. They were subjected to 120-min MCAO 2 weeks after the fecal transplantation and named Young-saline, Young-antibiotic, Young-oFMT and Young-yFMT, respectively. Young mice transplanted with old or young mouse feces but without MCAO were called Young-oFMT-Sham and Young-yFMT-Sham, respectively. A: body weights at baseline. B: body weights after the treatment of cefazolin. C: body weights 14 days after fecal transplantation. D: body weights of control mice and mice treated with cefazoline during the period from before to 21 days after the first dose of cefazolin treatment. E: cerebral blood flow (CBF) changes. F: colon mucosal permeability. Parametric results in normal distribution are in mean ± S.E.M. (panels C, D and F) and other results that are nonparametric data or parametric data in non-normal distribution are presented as median with interquartile range (all other panels). Data of each individual animal is also presented (n = 15 for panels A to C, n = 9 – 12 for panel D, n = 8 – 12 for panel E). Fig. S3. Determination of the dosage of valeric sodium used in the study. Young mice received two intraperitoneal 100 mg/kg valeric sodium in 100 μl or [file 40168_2023_1648_MOESM2_ESM.docx]

**Figure legends**

***Fig. S1:*** *Effects of gut microbiota transplant on the total bacterial load in the feces.* Young mice (8-weeks old) received transplantation of young mouse (8 – 10 weeks old) feces (Young-yFMT) or old mouse (18 – 21 months old) feces (Young-oFMT). Feces were harvested 14 days after the transplantation and the gut microbiota was analyzed. Top panel: a representative image of 16S rRNA PCR products. Bottom panel: quantification of the products. Results are in mean ± S.E.M. (n = 8).

***Fig. S2:*** *Body weight, cerebral blood flow decrease during middle cerebral artery occlusion and colon mucosal permeability of mice with or without fecal transplantation.* Young mice (8-weeks old) received saline, cefazolin, cefazolin and then transplantation of old mouse feces or cefazolin and then transplantation of young mouse feces. They were subjected to 120-min MCAO 2 weeks after the fecal transplantation and named Young-saline, Young-antibiotic, Young-oFMT and Young-yFMT, respectively. Young mice transplanted with old or young mouse feces but without MCAO were called Young-oFMT-Sham and Young-yFMT-Sham, respectively. A: body weights at baseline. B: body weights after the treatment of cefazolin. C: body weights 14 days after fecal transplantation. D: body weights of control mice and mice treated with cefazoline during the period from before to 21 days after the first dose of cefazolin treatment. E: cerebral blood flow (CBF) changes. F: colon mucosal permeability. Parametric results in normal distribution are in mean ± S.E.M. (panels C, D and F) and other results that are nonparametric data or parametric data in non-normal distribution are presented as median with interquartile range (all other panels). Data of each individual animal is also presented (n = 15 for panels A to C, n = 9 – 12 for panel D, n = 8 – 12 for panel E).

***Fig. S3:*** *Determination of the dosage of valeric sodium used in the study.* Young mice received two intraperitoneal 100 mg/kg valeric sodium in 100 μl or 100 μl saline at an interval of 7 h. Old mice receiving saline injection were also included. Their blood was harvested 3 h after the second injection for measuring valeric acid. Results are in mean ± S.E.M. Data of each individual animal is also presented (n = 8).


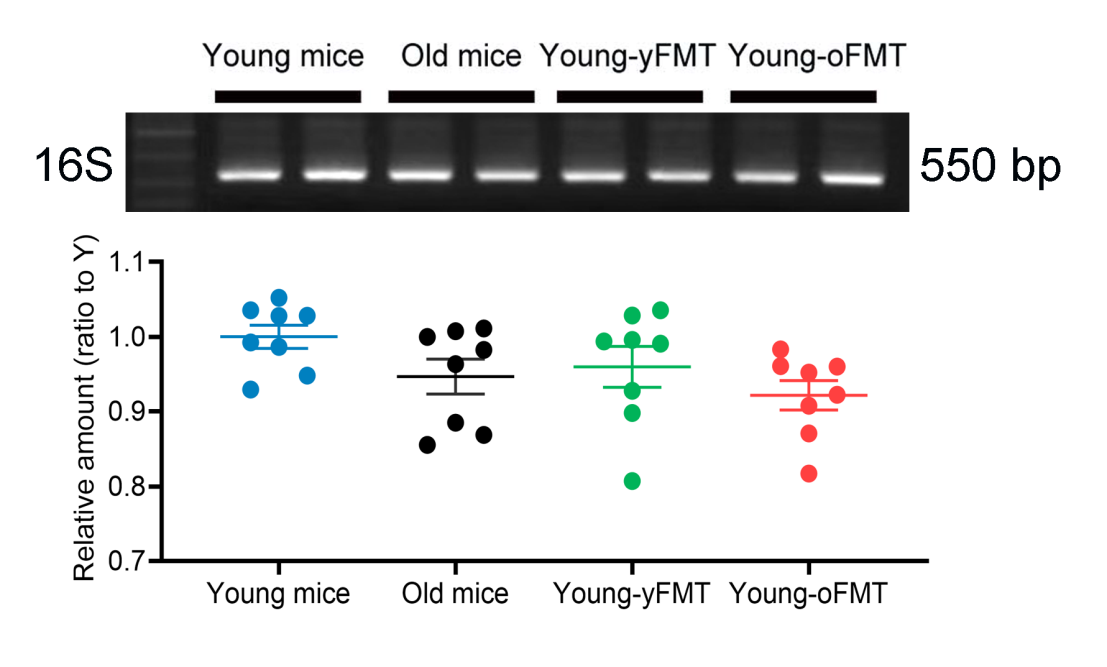


Fig. S1


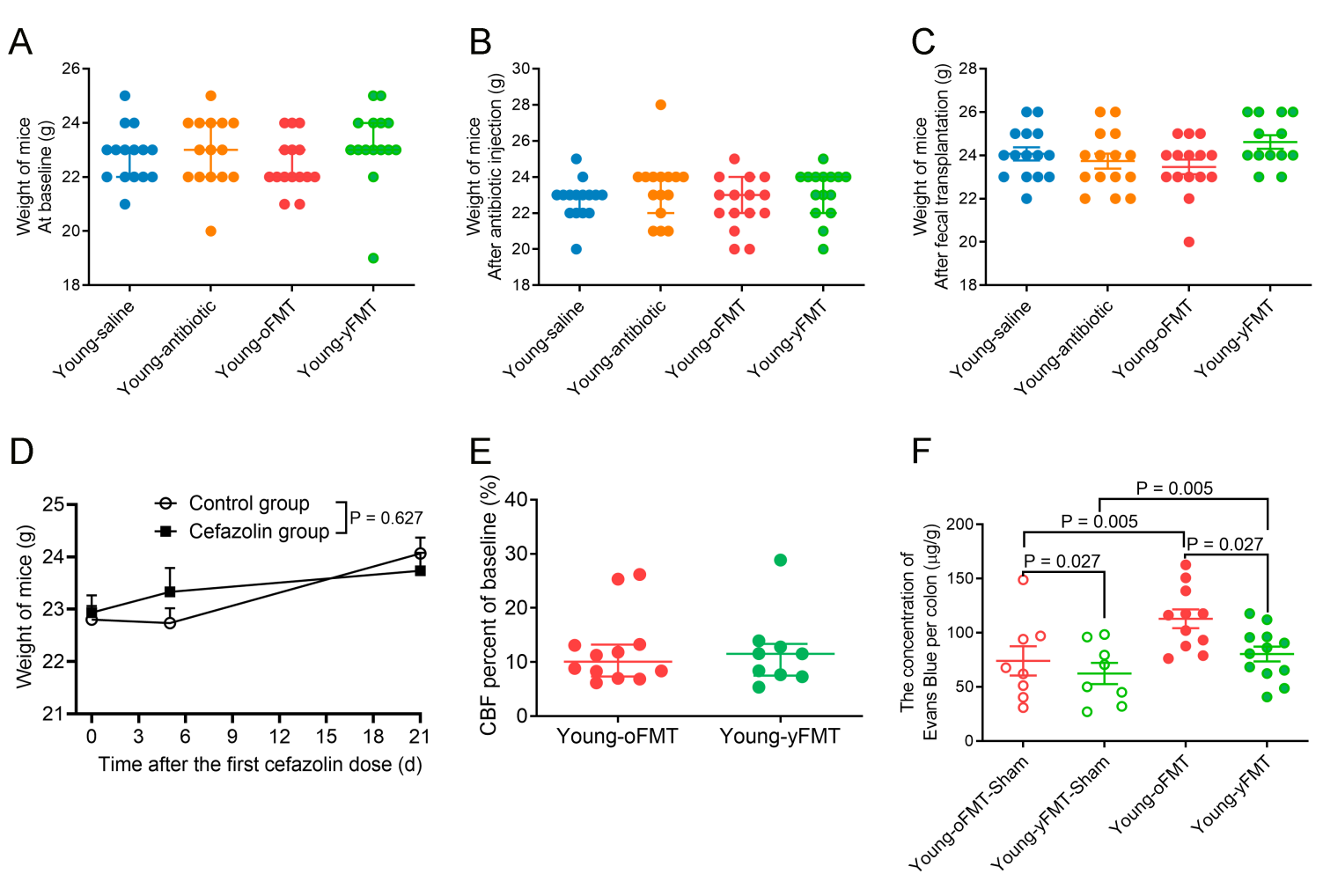


Fig. S2


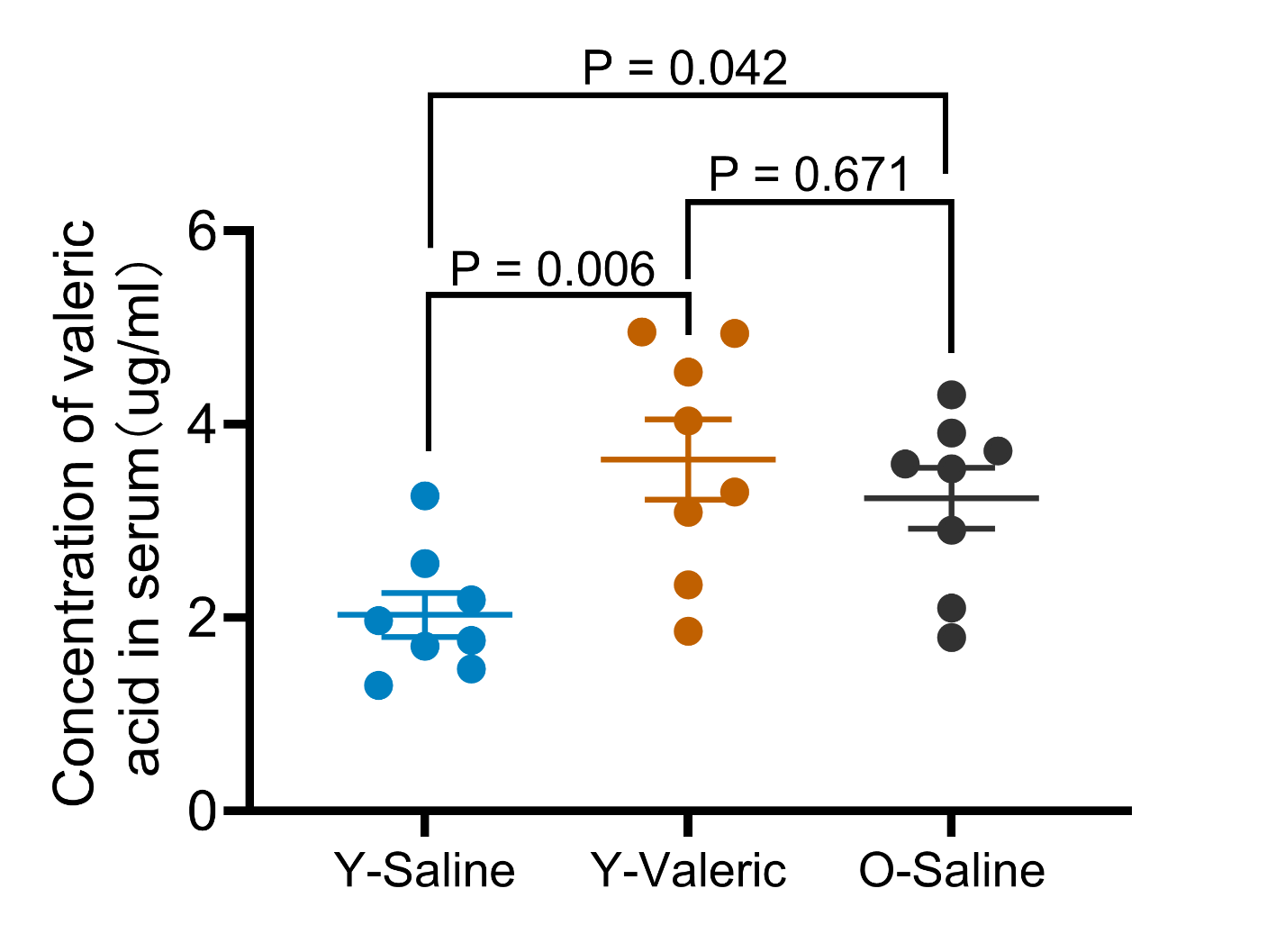


Fig. S3
